# Spirorchiidiasis in Stranded Olive Ridley Turtles (*Lepidochelys olivacea*) Along the Northern and Central-Northern Chilean Coast

**DOI:** 10.3390/ani16162492

**Published:** 2026-08-11

**Authors:** Carlos A. Flores Olivares, Juan Pablo Ruíz Yañez, Gerardo Cerda Gaete, Sofía Marambio Cortés, Tomás Pino Damke, Carlos Sandoval Hurtado, Constanza Rodríguez Latorre, Gerardo Tapia Miranda, Pablo Oyarzún-Ruiz

**Affiliations:** 1 Laboratorio de Ciencias Veterinarias, Universidad del Alba, La Serena 1700000, Chile; 2 Servicio Nacional de Pesca y Acuicultura, Coquimbo 1780000, Chile; 3 Conservación Humboldt, Non-Governmental Organization, Coquimbo 1780000, Chile; 4 Veterinary Histopathology Center, Puerto Montt 5480000, Chile; 5 Instituto de Patología Animal, Universidad Austral de Chile, Valdivia 5090000, Chile; 6 Servicio Nacional de Pesca y Acuicultura, Atacama 1490000, Chile; 7 Departamento de Microbiología, Facultad de Ciencias Biológicas, Universidad de Concepción, Concepción 4081407, Chile

**Keywords:** spirorchiid, blood flukes, *Lepidochelys olivacea*, digeneans, wildlife pathology

## Abstract

The olive ridley turtle (*Lepidochelys olivacea*) is the second smallest and most abundant of the seven sea turtle species worldwide, currently distributed across approximately 80 countries. However, due to various anthropogenic and environmental pressures, its populations are declining and categorized as “Vulnerable” by the IUCN. Postmortem macroscopic and microscopic examination detected spirorchiids and associated lesions in six adult female olive ridley turtles is described herein, with strandings occurring along the Northern and Central-Northern Chilean coast between September 2024 and January 2026. Gross findings included alterations in the gastrointestinal, reproductive, and vascular systems, characterized by transmural granulomatous lesions and mucosal thickening. Histopathological analysis confirmed the presence of these cryptic parasites in multiple organs, including the intestine, reproductive tract, vasculature, liver, spleen, and adrenal glands, with varying degrees of severity. These findings confirm the presence of spirorchiidiasis in olive ridley turtles in Chile, which may have contributed to stranding and mortality events in some individuals. Molecular characterization is essential to determine whether this agent corresponds to a known species or represents a previously unreported spirorchiid taxon in the Southeastern Pacific.

## Simple Summary

The olive ridley turtle (*Lepidochelys olivacea*) is classified as “Vulnerable” by the International Union for Conservation of Nature (IUCN); therefore, understanding the diseases affecting this species is essential for its conservation. This study provides the first evidence of spirorchiidiasis in olive ridley turtles from the Southeastern Pacific. All examined specimens exhibited lesions compatible with spirorchiidiasis, with varying degrees of severity affecting multiple organ systems. The most severe lesions involved the gastrointestinal and vascular systems, whereas milder alterations were observed in the reproductive system, spleen, liver, and adrenal glands. The pathological impact was classified as incidental, contributory, fatal, and unknown. The identification of these parasites and associated pathological alterations is crucial for assessing and improving health management protocols in rehabilitation and conservation centers for olive ridley turtles.

## 1. Introduction

The olive ridley turtle (*Lepidochelys olivacea*) is a circumtropical sea turtle species currently listed as “Vulnerable” by the International Union for Conservation of Nature (IUCN) [1]. Throughout its distribution, this species faces significant risks that lead to frequent stranding events, including habitat degradation and diverse anthropogenic pressures as highlighted in global and regional recovery assessments [2,3]. Specific threats affecting survival include predation of eggs and hatchlings by birds [4], domestic dogs [5], and large felids [6], as well as infectious diseases such as fibropapillomatosis [7]. Furthermore, environmental stressors, including heavy metal accumulation [8], oil pollution [9], vessel collisions [10], and other direct anthropogenic impacts [11], play a critical role in population declines worldwide. Beyond these traumatic and environmental causes, infectious diseases, including parasitic infections, are important determinants of health of these chelonians, often acting as determining or contributory factors in the mortality of stranded individuals [12]. Among the parasitic diseases affecting olive ridley turtles, infections caused by blood flukes of the Spirorchiidae family are recognized as a significant pathological condition and have been associated with mortality in some cases [13,14]. These endoparasites inhabit the cardiovascular system and are responsible for spirorchiidiasis, a multisystemic pathology characterized by the presence of adults in large vessels and the dissemination of eggs toward various vital organs [15]. The Spirorchiidae family comprises approximately 21 genera, 10 of which exclusively parasitize marine turtles, including the olive ridley turtle [16]. Although spirorchiidiasis is considered near cosmopolitan, it has been documented in only five of the seven marine turtle species, with the pathology and host specificity being topics of active molecular and clinical research [15,17]. Host inflammatory responses to these embolized eggs can trigger chronic granulomatous inflammation that can severely compromise tissue function in multiple systems [18]. In severe cases, this infection leads to arteritis, thrombosis, and systemic debility, which may cause the direct death of the animal [12]. Despite the importance of this parasitosis, reports in the Southeastern Pacific are extremely limited. While lesions associated with Spirorchiidae have been described in olive ridley turtles and green turtles (*Chelonia mydas*) in the South Atlantic (Brazil) [19] and Central America (Costa Rica) [20], there are no previous records documenting the impact of these trematodes on sea turtles in the South-Eastern Pacific.

The intravascular localization of blood flukes often hinders their detection, particularly in regions where systematic monitoring is limited. The National Fisheries and Aquaculture Service of Chile (SERNAPESCA) conducts constant monitoring of strandings, but the histopathological characterization of internal parasitic diseases remains an area with critical knowledge gaps regarding the prevalence and diversity of these etiological agents [21]. Therefore, the objective of this communication is to report, for the first time, the presence of histopathological lesions associated with spirorchiidiasis in six adult female olive ridley turtle specimens stranded along the Northern and Central-Northern Chilean coast between 2024 and 2026. This study describes the multisystemic distribution of lesions, evaluates the impact of infection on the health and mortality of these individuals, and contributes to the epidemiological knowledge of these parasites in the South Pacific.

## 2. Materials and Methods

### 2.1. Stranding Records and Sample Collection

**Figure 1:**
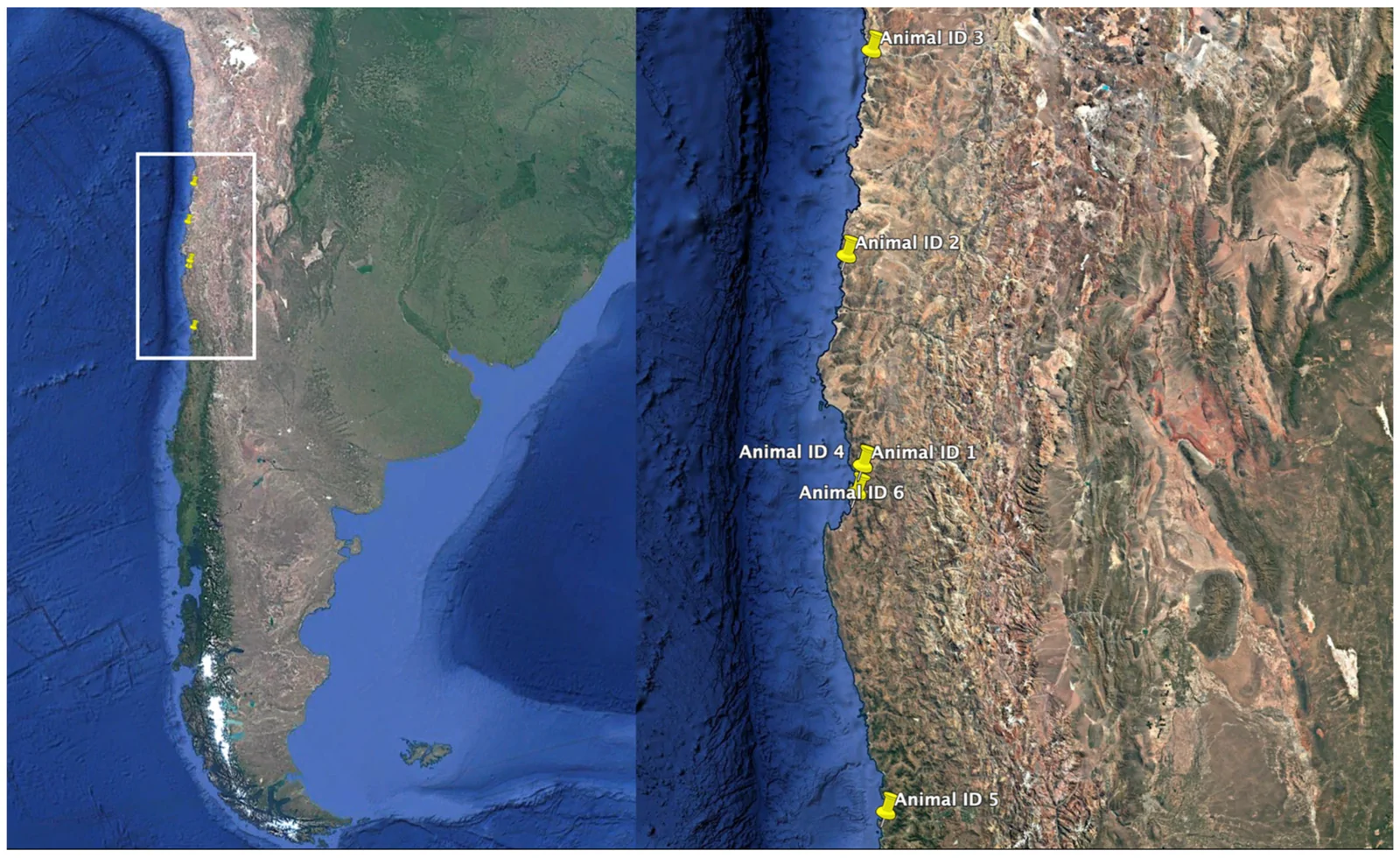
Map indicating the stranding locations of adult olive ridley turtles along the Northern and Central-Northern Chilean coast between September 2024 and January 2026, with evidence of spirorchiidiasis (Base map generated using Google Earth Pro, version 7.3.7.1155).

Since the initiation of this study along the Chilean coast, a total of 26 stranded olive ridley sea turtles were reported by the SERNAPESCA. Only six carcasses met the freshness criteria required for complete pathological examination and histopathological sampling. Of these individuals, six adult females were available for necropsy and donated for teaching and research purposes. These turtles stranded either alive (*n* = 4) or were found dead (*n* = 2) along the northern (Atacama) and central-northern (Coquimbo and Valparaíso) coasts of Chile between 2024 and 2026 (Figure 1). These stranding events occurred across three administrative regions: Atacama, Coquimbo, and Valparaíso. Geographic coordinates were plotted using Google Earth Pro (Google LLC, Mountain View, CA, USA) to generate the distribution map. All specimens were donated for scientific research under official authorizations issued by the National Fisheries and Aquaculture Service (SERNAPESCA) for the Coquimbo and Atacama regions, including one animal originating from Valparaíso that was referred to and authorized for diagnostic evaluation through SERNAPESCA Coquimbo. Data recorded for each individual included identification number, stranding condition (live or dead), body condition, stranding date, date of death, and geographic location. Body condition score (BCS) was determined following the criteria established by Stacy et al. [12].

### 2.2. Necropsy and Histopathology

Complete necropsies were performed at the Laboratory of Veterinary Science Research (LiCiVet), Universidad del Alba, La Serena, Chile. A comprehensive gross examination of all organs was conducted, and representative tissue samples from the gastrointestinal tract, liver, reproductive tract, heart, adrenal glands, spleen, kidneys, and major blood vessels were collected and fixed in 10% neutral buffered formalin (DiaPath, Martinengo, Italy). These fixed tissues were submitted to the Veterinary Histopathology Center (VeHiCe) in Puerto Montt, Chile, for routine histological processing. Tissue sections (4 µm) were stained with hematoxylin and eosin (H&E), Gram, Periodic acid–Schiff (PAS) and Ziehl–Neelsen stains and subsequently digitized using a Motic Easy Scan system (Motic, Xiamen, China).

### 2.3. Lesion Grading and Spirorchiid Impact Rating

Based directly on the methodology described by Stacy et al. (2010) [12], vascular damage, specifically arteritis, was evaluated through integrated gross and microscopic findings and categorized into four distinct grades. Grade 0 was defined by the absence of gross and microscopic lesions. Grade 1 included focal lesions less than 1.5 cm in diameter with histological evidence of minimal inflammation and/or fibrosis. Grade 2 comprised focally extensive lesions between 1.5 and 4 cm in diameter, or a maximum of three lesions, characterized microscopically by moderate inflammation and intimal proliferation. Grade 3 was assigned to focally extensive or multiple lesions greater than 4 cm in dimension or those involving one or more major blood vessels, with histological criteria including thrombi or medial necrosis. Enteric spirorchiidiasis was similarly graded based on the microscopic identification of distinct egg masses: Grade 0 (no lesions), Grade 1 (1–10 egg masses), Grade 2 (more than 10–50 egg masses), and Grade 3 (more than 50 egg masses). Based on these integrated pathological findings and concurrent clinical data, the final impact rating was classified as none detected, incidental infection (low parasite load and mild inflammation), contributory (significant parasite burden associated with complications such as thrombosis or secondary infections), fatal (severe lesions directly linked to mortality), or unknown, where significant damage was present but the definitive cause of death could not be determined due to concurrent factors [12,22].

## 3. Results

### 3.1. Stranding Records and Biological Data

A total of six olive ridley turtles were analyzed, all of which were identified as adult females. The strandings occurred between September 2024 and January 2026 across the regions of Atacama (*n* = 2), Coquimbo (*n* = 3), and Valparaíso (*n* = 1). Body condition was classified as intermediate or poor. Two specimens were found dead on arrival and were delivered directly by SERNAPESCA from the Atacama and Coquimbo regions. The four turtles that stranded alive were transferred to the Humboldt Conservation Rescue Center (Coquimbo Region) for general clinical examination and quarantine (Table 1).

### 3.2. Postmortem Findings

Postmortem examination of the six specimens revealed multisystemic lesions compatible with spirorchiidiasis.

#### 3.2.1. Gross Findings

**Figure 2:**
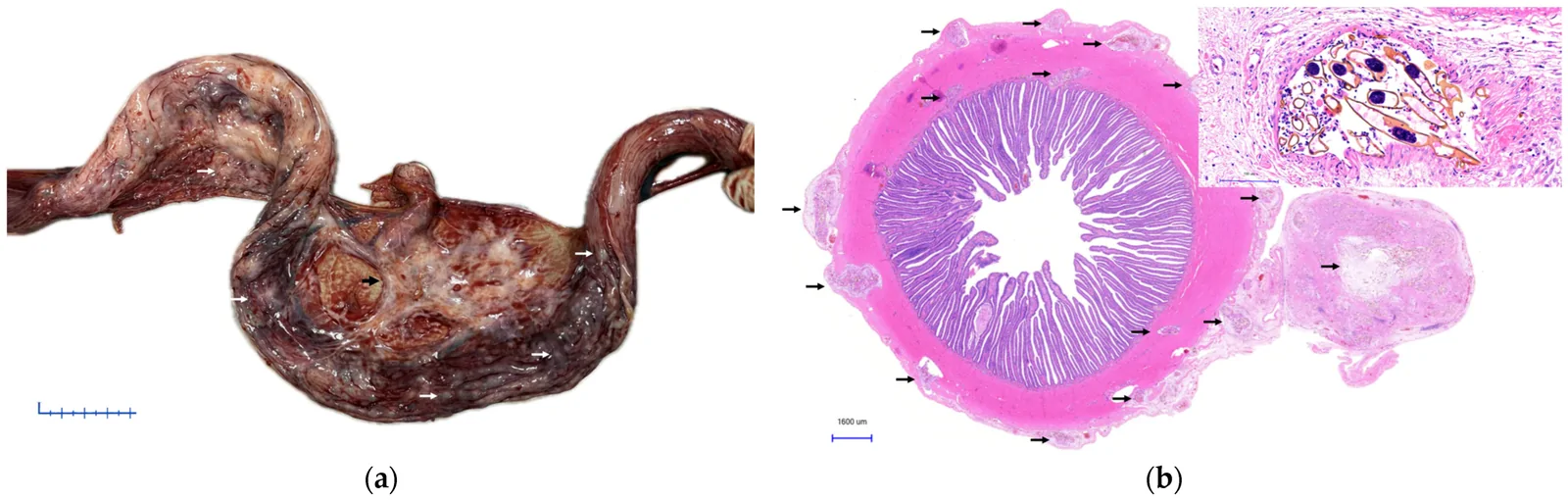
Duodenal lesions associated with spirorchiids in an adult female olive ridley turtle stranded in Chile (Animal ID 3). (**a**) Gross appearance: Thickened, indurated duodenal walls with multiple transmural granulomatous lesions (white arrows) and lymphangiectasia (black arrow). Bar = 4 cm. (**b**) Multifocal fibrosing granulomas are present in the mucosa, submucosa, muscularis, and adjacent tissue (black arrows). Bar = 1600 µm. Inset: Large-caliber blood vessel containing abundant trematode eggs and miracidia. The eggs measured up to 180 µm in length and 25 µm in width. (H&E). Bar = 100 µm.

**Figure 3:**
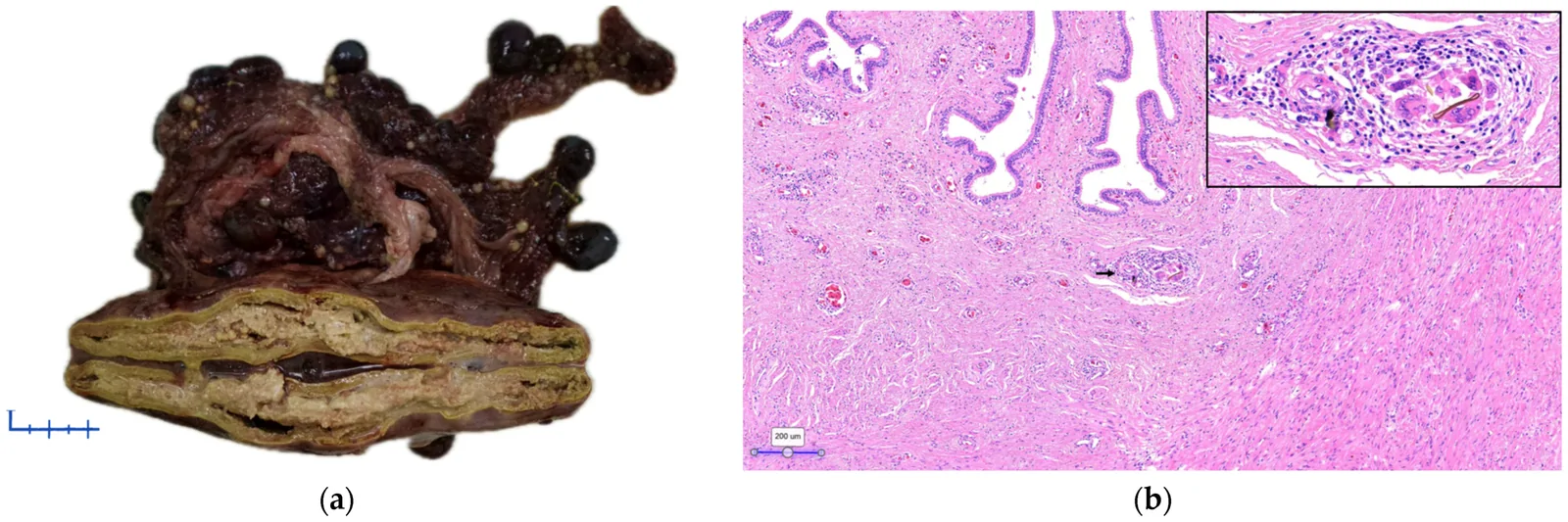
Oviduct lesions associated with spirorchiids in an adult female olive ridley turtle stranded in Chile (Animal ID 3). (**a**) Gross appearance: Mature oviduct adjacent to the ovary, which contains follicles and corpora albicantia, with multifocal fibrosing granulomas. The section reveals a granulomatous appearance with a whitish-to-yellowish center. Bar = 2 cm. (**b**) Micrograph of the oviduct showing a fibrosing granulomatous lesion (black arrow). Inset: Lymphohistiocytic to granulomatous inflammation with multinucleated giant cells surrounding trematode eggs. (H&E). Bar = 200 µm.

**Figure 4:**
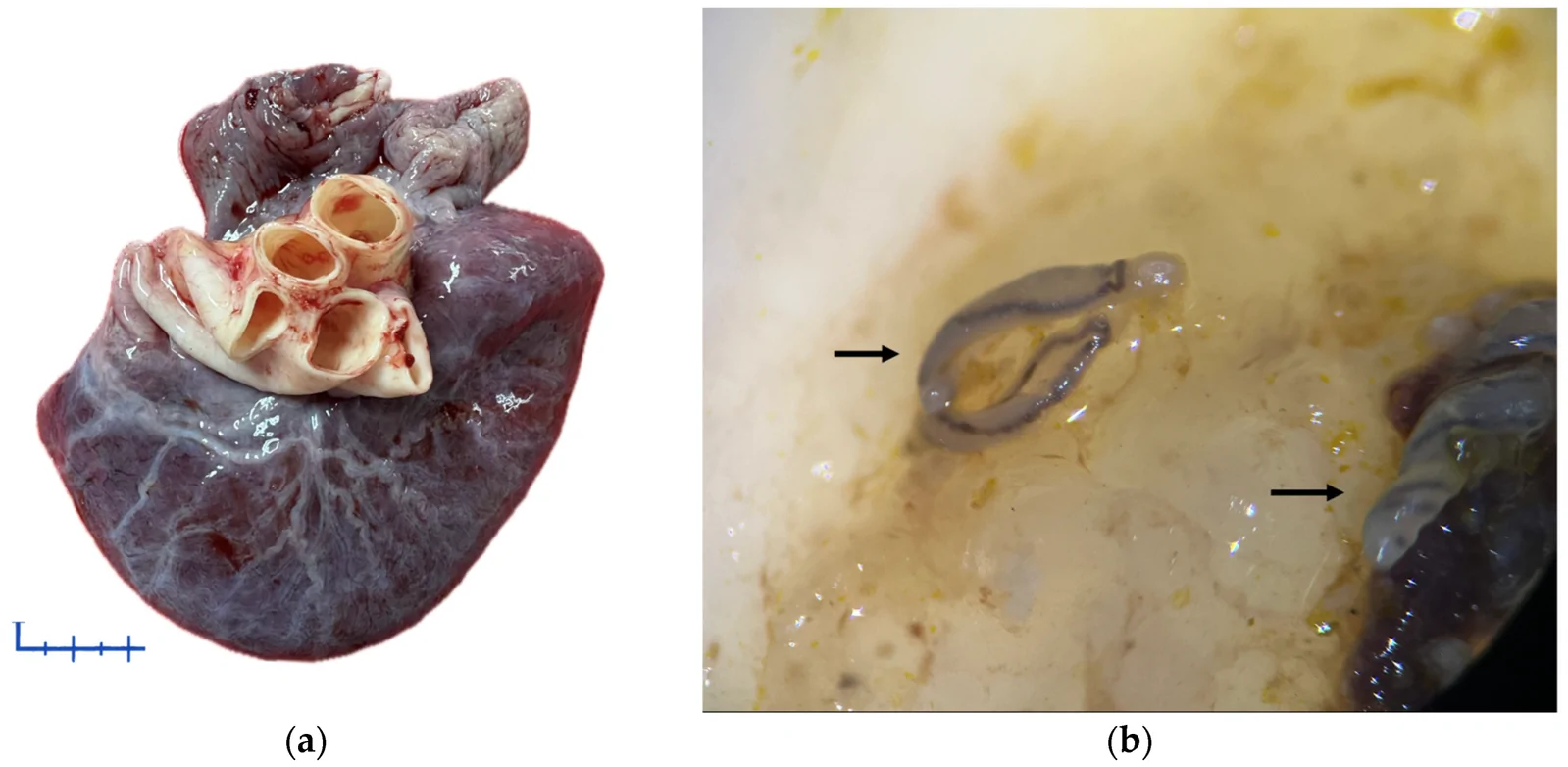
Heart and great vessels of an adult female olive ridley turtle stranded in Chile (Animal ID 2) showing lesions associated with spirorchiids. (**a**) Heart with serous atrophy of fat and lymphangiectasia. Bar = 2 cm. (**b**) Adult spirorchiid trematodes within the lumen of a large-caliber artery (arrows).

All necropsies were performed between 2 and 12 h after death had been confirmed by official authorities. All turtles exhibited marked gastrointestinal alterations characterized by varying degrees of mucosal thickening and intestinal edema. Multifocal to coalescing transmural granulomatous lesions were observed in the subserosa, with the largest masses measuring up to 10 × 12 × 7 cm (Figure 2a). In the liver, multifocal nodular granulomatous lesions were identified in three specimens (Animal IDs 3, 4, and 6). Specifically, Animal ID 3 presented a large granulomatous lesion (20 × 10 × 6 cm) adjacent to a mature oviduct; upon sectioning, the mass exhibited a friable, whitish-to-yellowish appearance (Figure 3a). Cardiac examination revealed serous atrophy of fat and lymphangiectasia (Figure 4a). Vascular evaluation confirmed the presence of adult trematodes within the lumen of major vessels (Figure 4b). Adult specimens were preserved in 70% ethanol and frozen for subsequent molecular analyses. Morphologically, the parasites were consistent with trematodes of the family *Spirorchiidae*, observed either attached to the vascular endothelium or forming intravascular aggregates. Concurrent findings included a severe cranial fracture and predator-inflicted bite wounds in Animal ID 2, and an embedded fishhook within the esophageal mucosa of Animal ID 3, associated with chronic fibrous tissue encapsulation.

#### 3.2.2. Microscopic Findings, Lesion Grading and Spirorchiid Impact Rating

The most relevant findings consisted of trematode eggs located at the center of granulomas in multiple anatomical sites, characterized by an elongated yellow-brown shell, with miracidia occasionally observed within the eggs. In the intestine, duodenal villi were markedly thickened due to dense lymphoplasmacytic inflammatory infiltrates in the lamina propria. Numerous elongated eggs compatible with members of the *Spirorchiidae* family were observed, often surrounded by histiocytes and multinucleated giant cells (MGCs). Multifocal to coalescing necrotizing and fibrosing granulomas of variable diameter were distributed throughout the submucosa, muscularis, and serosa; these lesions featured necrotic centers containing parasite eggs and, in some cases containing developed miracidia (Figure 2b). Vascular lesions included the formation of parasitic thrombi and emboli. Affected vessels showed marked intimal proliferation, medial necrosis, and adventitial thickening. In animal IDs 4 and 6, high egg concentrations within the vascular walls triggered severe endarteritis. Extra-vascular involvement included granulomas and MGCs surrounding spirorchiid eggs in the oviduct (Figure 3b), spleen (Animal IDs 4 and 6), and adrenal glands (Animal ID 2). Histopathological evaluation revealed variable degrees of vascular and gastrointestinal damage associated with spirorchiidiasis. According to the criteria proposed by Stacy et al. (2010) [12], the pathological impact was classified into different categories, and these findings are summarized in Table 2. Gram, PAS, and Ziehl–Neelsen stains were negative in all tissue samples examined. No Gram-positive or Gram-negative bacteria, fungal organisms, or acid-fast microorganisms were identified in any of the analyzed specimens.

## 4. Discussion

The findings presented in this study constitute the first formal report of lesions associated with spirorchiid trematodes, including both eggs and adult parasites, in olive ridley turtles from the Southeastern Pacific region with the presence of eggs and adult parasites. While spirorchiidiasis is considered a near-cosmopolitan disease, it has been documented in only five of the seven marine turtle species, with no confirmed reports to date in the leatherback (*Dermochelys coriacea*) or the flatback (*Natator depressus*) turtles [15]. In olive ridley turtles, lesions associated with spirorchiid eggs have been reported along the Atlantic coast of Brazil [19] and the Pacific coast of Costa Rica [20]. However, evidence of both adult spirorchiids and eggs in olive ridley turtles has only been reported in Australia, where molecular analyses of the detected species have also been conducted [23]. The findings presented herein expand the documented geographical distribution of these parasites in the Pacific, suggesting a wider presence in temperate latitudes than previously documented. The most characteristic presentation of spirorchiidiasis is chronic granulomatous inflammation around embolized eggs, a finding observed in all the cases analyzed in this study. According to Chapman et al. [15], the organs most frequently affected by egg-associated granulomas include the lungs, spleen, gastrointestinal tract, and thyroid gland. The results obtained in this study align with these observations, particularly regarding the high prevalence of gastrointestinal system involvement. While the thyroid gland is often reported as a common site of infection in loggerheads (*Caretta caretta*) and green turtles, the documentation of granulomas in the adrenal glands (Animal ID 2) and oviduct (Animal ID 3) in the present study is a notable finding. The presence of eggs in the adrenal glands could interfere with the host’s endocrine response to stress, while oviduct involvement might impact reproductive health, highlighting the high invasive potential of these parasites in olive ridley turtles [12,18]. Species of the genus Hapalotrema, previously reported from the South Atlantic and Australia, are considered highly pathogenic turtle blood flukes and have been associated with severe vascular lesions [23,24]. Evaluating the impact of spirorchiidiasis on stranding events requires distinguishing between incidental, contributory, and fatal infections. In this study, infection was considered fatal in Animal ID 6 based on the severity of vascular occlusion and subsequent severe multisystemic lesions, with no gross or microscopic evidence of other pathological alterations that could be associated with the cause of death. In Animal IDs 2, 3, and 5, the pathological significance of spirorchiidiasis was considered uncertain because alternative causes of death or major contributing factors were identified. These findings are consistent with a cumulative stress hypothesis that chronic infections by these cryptic parasites cause systemic debility, increasing host vulnerability to traumatic events or secondary infections [15]. In the present cases, severe lesions associated with spirorchiid trematode infections were observed, occasionally accompanied by granulomatous inflammation. No evidence of infectious agents was identified using ancillary diagnostic techniques. Similar pathological findings have been reported in several sea turtle species and have been associated with a variety of infectious agents, including parasites, bacteria, and fungi. However, it remains unclear whether these co-infections exacerbate disease severity and contribute to mortality or merely represent incidental findings [13,25,26,27].

For definitive confirmation and species-level identification of these parasites, molecular techniques remain essential due to the high level of diversity within the family Spirorchiidae [17]. To contextualize these findings within the regional and global conservation of sea turtles, it is relevant to consider the ecology of these species in Chile [28]. Furthermore, these results complement pathological records from other regions such as Indonesia [29], Australia [30], Taiwan [31], and Brazil [32]. The pathological patterns observed here are consistent with lesions described in other species, such as green turtles in the Caribbean [33] and the Mediterranean [34], where specialized qualitative and quantitative methods are increasingly utilized to estimate parasite burden and health impact [22]. Future research in the Southeastern Pacific should prioritize the integration of these advanced diagnostic tools with systematic necropsies to clarify the species composition, transmission dynamics, and true clinical significance of spirorchiids in this region. Additionally, molecular analyses and the development of morphological identification keys for spirorchiid species identified in Latin America are needed to improve taxonomic resolution and epidemiological understanding.

## 5. Conclusions

This communication provides new information on trematode infections in olive ridley sea turtles from the southeastern Pacific, representing the first evidence of spirorchiidiasis in this host species in the region and expanding the documented distribution of these parasites into temperate latitudes. The histopathological findings, including multisystemic lesions involving the adrenal glands and oviduct, broaden the current understanding of the tissue distribution and pathological manifestations associated with spirorchiid infections in olive ridley sea turtles. Although based on a limited number of cases, the findings suggest that spirorchiidiasis may contribute to morbidity in stranded olive ridley sea turtles along the northern and central-northern coast of Chile, either as a primary pathological process or in combination with other natural or anthropogenic factors. These observations highlight the importance of considering spirorchiidiasis as a differential diagnosis during postmortem examinations of stranded sea turtles. The lesions documented in this study support the inclusion of systematic histopathological and molecular investigations in sea turtle stranding response programs to improve the detection and characterization of spirorchiid infections. Future studies incorporating larger sample sizes and molecular characterization of parasites are needed to clarify the diversity, distribution, and pathological significance of Spirorchiidae in the southeastern Pacific, thereby providing a stronger evidence base for the conservation and health assessment of vulnerable marine turtle populations.

## Figures and Tables

**Table 1:** Data from adult female olive ridley turtles stranded along the Northern and Central-Northern Chilean coast between September 2024 and January 2026 with evidence of spirorchiidiasis.

Animal ID	Stranding Report	Stranding Condition	Body Condition Score	Stranding Date	Date of Death	Geographical Location	Region
1	13,066	Alive	Intermediate	21 September 2024	22 September 2024	29°55′29″ S, 71°16′43″ W	Coquimbo
2	14,911	Dead	Poor	3 July 2025	Dead on arrival	28°09′54″ S, 71°10′09″ W	Atacama
3	14,427	Alive	Intermediate	3 November 2025	4 November 2025	26°27′50″ S, 70°41′12″ W	Atacama
4	14,395	Alive	Poor	4 November 2025	5 November 2025	29°55′12″ S, 71°16′37″ W	Coquimbo
5	14,521	Alive	Poor	19 December 2025	20 December 2025	32°50′19″ S, 71°31′18″ W	Valparaíso
6	14,613	Dead	Poor	2 January 2026	Dead on arrival	30°08′35″ S, 71°22′23″ W	Coquimbo

**Table 2:** Characterization of vascular and enteric lesions associated with spirorchiidiasis, assessment of pathological impact, and determination of cause of death in stranded adult female olive ridley turtles according to the criteria proposed by Stacy et al. (2010) [12].

Animal ID	Vascular Damage	Enteric Spirorchiidiasis	Impact Rating	Cause of Death
1	1	1	Incidental Infection	Unknown
2	2	2	Unknown	Severe Cranial fracture
3	2	3	Unknown	Esophageal Fishhook
4	2	3	Contributory	Unknown
5	1	2	Incidental Infection	Unknown
6	3	3	Fatal	*Spirorchiidiasis*

## Data Availability

The raw data supporting the conclusions of this article will be made available by the authors on request.

## Abbreviation

The following abbreviation is used in this manuscript:

IUCN	International Union for Conservation of Nature
